# Supplementary material for: Splice-Junction-Based Mapping of Alternative Isoforms in the Human Proteome
Source: Cell Rep. Author manuscript; Available in PMC 2020 Jan 15. (PMC6961840; doi:10.1016/j.celrep.2019.11.026)
Supplement: 2 [file NIHMS1546469-supplement-2.zip › DF1/PXD010154/Thyroid/Thyroid_15_TG_TVAQNNAPSFCPLVVLPSLTEK.pdf]

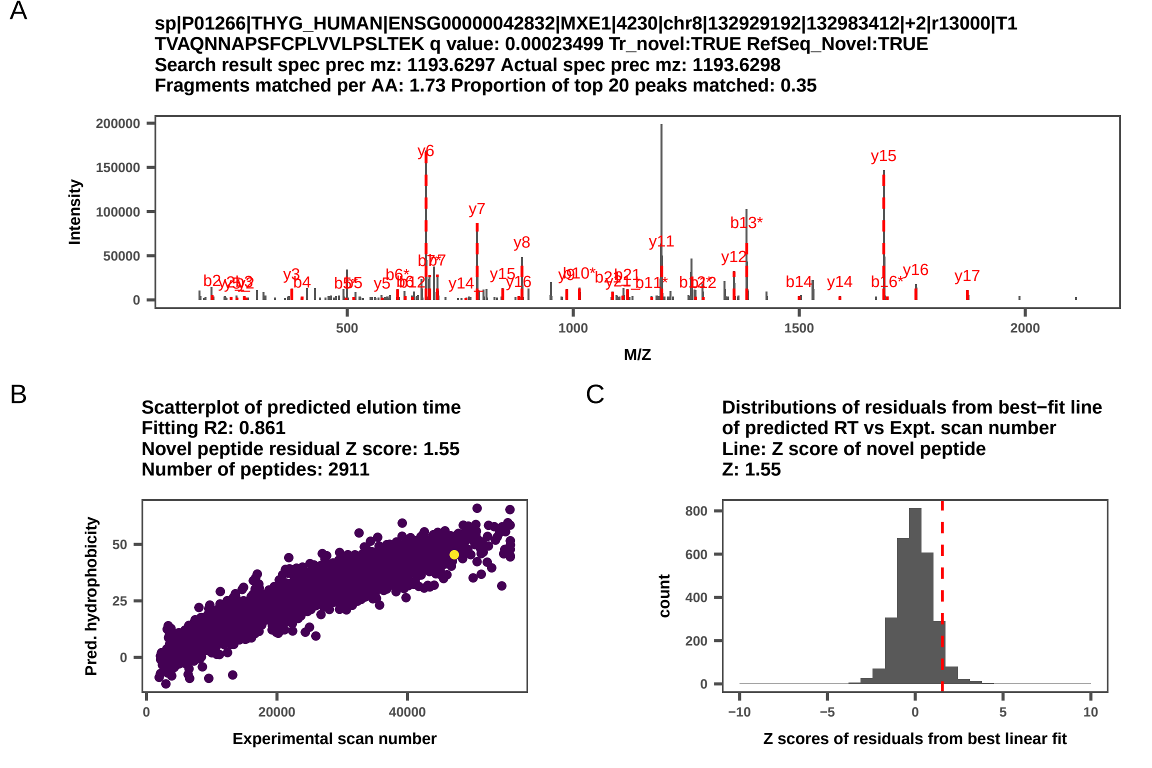
A
sp|P01266|THYG_HUMAN|ENSG00000042832|MXE1|4230|chr8|132929192|132983412|+2|r13000|T1
TVAQNNAPSFCPLVVLPSLTEK q value: 0.00023499 Tr_novel:TRUE RefSeq_Novel:TRUE
Search result spec prec mz: 1193.6297 Actual spec prec mz: 1193.6298
Fragments matched per AA: 1.73 Proportion of top 20 peaks matched: 0.35
Intensity
200000
150000
100000
50000
0
500
1000
1500
2000
M/Z
y6
y15
y7
b13*
y8
y11
y12
y16
y17
y3
b4
b14
y14
B
Scatterplot of predicted elution time
Fitting R2: 0.861
Novel peptide residual Z score: 1.55
Number of peptides: 2911
Pred. hydrophobicity
50
25
0
20000
40000
Experimental scan number
C
Distributions of residuals from best−fit line
of predicted RT vs Expt. scan number
Line: Z score of novel peptide
Z: 1.55
count
800
600
400
200
0
−10
−5
0
5
10
Z scores of residuals from best linear fit
